# A Narrative Review of the Safety of Anti-COVID-19 Nutraceuticals for Patients with Cancer

**DOI:** 10.3390/cancers13236094

**Published:** 2021-12-03

**Authors:** Karlen Stade Bader-Larsen, Elisabeth Anne Larson, Maria Dalamaga, Faidon Magkos

**Affiliations:** 1 Department of Nutrition, Exercise and Sports, University of Copenhagen, 1958 Frederiksberg, Denmark; skc639@alumni.ku.dk (K.S.B.-L.); kfg323@alumni.ku.dk (E.A.L.); 2 Department of Biological Chemistry, Medical School, National and Kapodistrian University of Athens, 11527 Athens, Greece; madalamaga@med.uoa.gr

**Keywords:** cancer, nutraceuticals, supplements, COVID-19, SARS-CoV-2

## Abstract

**Simple Summary:**

Dietary supplement use has increased more than 35% globally since the COVID-19 outbreak. While some nutraceuticals are potentially efficacious against severe disease from COVID-19, their indiscriminate use by patients with cancer without medical supervision is concerning. The aim of this narrative review was to evaluate the data on safety of “anti-COVID-19” nutraceuticals for patients with cancer. We found that the use of vitamin C, vitamin D, and selenium supplements is likely safe and even potentially beneficial at typically recommended doses. However, caution is advised regarding the use of omega-3 fatty acids and zinc, as risks from their use may outweigh the benefits.

**Abstract:**

Interest in dietary supplements and their efficacy in treating and preventing disease has increased greatly since the outbreak of the COVID-19 pandemic. Due to the risk of severe COVID-19 in patients with cancer, we conducted a narrative review aiming to better understand the data on the safety of the most efficacious “anti-COVID-19” nutraceuticals for patients with cancer. We conducted a PubMed database search aimed at identifying the most effective nutrients for use against COVID-19. For the identified nutraceuticals, we searched PubMed again regarding their safety for patients with cancer. Fifty-four total records (52 independent studies) were retrieved, pertaining to vitamin D, vitamin C, selenium, omega-3 fatty acids, and zinc. Vitamin D results from 23 articles indicated safe use, but two articles indicated potential harm. All 14 articles for vitamin C and five out of six articles for selenium indicated the safety of use (one study for selenium suggested harm with high-dose supplementation). Results for omega-3 fatty acids (seven articles) and zinc (one article), however, were rather mixed regarding safety. We conclude that vitamin D, vitamin C, and selenium supplements are likely safe or even beneficial at typically recommended doses; however, caution is urged with omega-3 fatty acid supplements, and zinc supplements should likely be avoided. More experimental research is needed, and nutraceutical use by patients with cancer should always be under the supervision of a healthcare team.

## 1. Introduction

In December 2019, a novel virus of unknown etiology was detected in Wuhan, China [1]. The virus, which most often manifests as a severe respiratory syndrome, quickly spread from Wuhan, with cases appearing globally by 30 January 2020 [2]. It was quickly labeled by the World Health Organization as a public health outbreak of international concern and was later declared a pandemic [2,3]. This novel airborne pathogen, since named severe acute respiratory syndrome coronavirus 2 (SARS-CoV-2), causes the disease now known as COVID-19 [2]. Despite containment efforts and the introduction of a vaccine in late 2020, by November 2021, over 5 million deaths had been attributed to the virus [4].

COVID-19 has been shown to manifest heterogeneously across different patient populations. Mild cases often result in flu-like symptoms, fever, or loss of taste and smell [5]. However, in severe cases, the effects of infection are more significant, resulting in an abnormal cytokine and chemokine response that causes systemic inflammation, affecting multiple tissues and organ systems [6]. Individuals with co-morbidities such as obesity, diabetes, cardiovascular disease, and cancer have a greater tendency to elicit this cytokine storm, making infection with COVID-19 particularly dangerous for these at-risk subgroups of the population [7,8,9,10,11].

Accordingly, attention has focused on protecting these vulnerable individuals, as well as the general public, from infection. However, the lack of efficacious pharmacological treatments for COVID-19 has led the public to seek alternative therapies, including nutraceuticals [12,13]. Nutraceuticals are foods or substances derived from food that may have a physiological effect or protect against disease. They have received heightened interest as some may affect the severity of COVID-19. For example, several observational studies have been published describing the association between specific nutrient deficiencies and COVID-19 severity and mortality [14,15]. A review by Vassiliou et al., which examined the role of vitamin D status in predicting outcomes in critical illness, concluded that there is an association between insufficient vitamin D status and infection, severity of illness, and mortality from COVID-19 [16]. Another review by Lordan et al. found an association between zinc deficiency and increased COVID-19 complications [17]. In vivo studies have also pointed to the role of nutraceuticals in the treatment and prevention of COVID-19, including a study by Corrao et al., which demonstrated an inverse relationship between C-reactive protein (CRP), a marker of systemic inflammation, and supplementation with vitamin C, vitamin D, and zinc [18]. Furthermore, there have been several theoretical papers discussing the potential mechanistic roles of nutraceuticals and how they might target the SARS-CoV-2 virus [19,20,21]. For example, for probiotics, one of the proposed mechanisms is by acting as angiotensin-converting enzyme (ACE) inhibitors, preventing SARS-CoV-2 from binding to ACE receptors in gastrointestinal cells [22]. For the keto-carotenoid astaxanthin (a terpene), it has been suggested that it may play a role in regulating reactive oxygen species formation, and therefore, supplementation may inhibit oxidative stress caused by SARS-CoV-2 [23]. Additionally, immunomodulatory nutraceuticals, such as glycophosphopeptide AM3, may be beneficial as either prophylactic or adjuvant therapy for SARS-CoV-2, as they improve the efficacy of action of natural killer cells and increase the production of anti-inflammatory cytokines [24]. While the use of most of these nutraceuticals is advocated on the basis of in vitro and in vivo observations for other similar viruses (e.g., SARS-CoV and MERS-CoV), there is a growing number of observational studies and randomized controlled trials (RCTs) specifically for COVID-19 that point to the potential efficacy of nutraceuticals in the fight against this novel pathogen.

The potential use of nutraceuticals for the supportive treatment of COVID-19 is particularly relevant and promising for those who are more susceptible to both infection and a severe course of the disease. Patients with cancer, in particular, may be at high risk of severe disease and mortality from COVID-19 depending on their disease stage, treatment, and type of cancer [25]. Generally speaking, there are numerous mechanisms behind the increased risk of COVID-19 infection in these patients, including immunosuppression from cancer therapy and immunosuppression from cancer itself [26]. Chemotherapy, which limits the growth of cancer cells, also impacts the production of white blood cells, leaving patients more susceptible to infection [27]. Patients with late-stage cancer are also at increased risk of infection as bone metastases can trigger an immune response that leads to bone marrow aplasia, resulting in a reduction of white blood cells, red blood cells, and platelets, which again leaves these individuals vulnerable to worse outcomes if infected with COVID-19 [28]. Additionally, patients with cancer tend to be older and have more co-morbidities, putting them at risk of a severe course of disease with COVID-19 [26].

It is therefore not surprising that the COVID-19 pandemic also resulted in increased fear and worsened anxiety and depression associated with a cancer diagnosis [29]. As such, many individuals, immunocompromised and healthy alike, have sought out ways to improve immunity [30]. Concurrently, popular media outlets have promoted the use of a variety of dietary supplements with putative immune-boosting potential that may help against COVID-19 infection [31,32]. This has led to a major increase in dietary supplement use during the pandemic, with a roughly 35% increase in North and South America, a 40% increase in Asia, and a 38% increase in Europe [30,33]. Concerningly, only 40% of these individuals consume supplements at the recommendation of a licensed medical professional [30].

Increased supplement use during the COVID-19 pandemic, especially without appropriate medical supervision, is troubling for oncologists and other oncology specialists. Specifically, one concern relates to the potential dampening of the cytotoxicity of chemotherapy by antioxidants and other supplements. The Diet, Exercise, Lifestyle, and Cancer Prognosis (DELCaP) study, a correlative study to the phase III SWOG SO221 [34], examined supplement use in patients with breast cancer and survivorship. This study found that the use of any antioxidant supplements, before or during breast cancer treatment, was associated with an increased risk of breast cancer recurrence and that vitamin B12 use during treatment was associated with poorer survival rates and poorer disease-free survival [34]. Results such as these indicate that nutraceutical use during or around chemotherapy may not be benign.

Given the rise in oral supplement use during the COVID-19 pandemic, as well as the increased interest in the efficacy of nutraceuticals in preventing or reducing the severity of COVID-19, we conducted a narrative review focusing on the safety of the most efficacious “anti-COVID-19” oral supplements for patients with cancer. As COVID-19 is still a present threat, individuals with cancer and their providers need up-to-date, evidence-based guidance for supplement use around their respective treatments.

## 2. Methods

We conducted our initial literature search on 8 September 2021 focusing on the efficacy of nutraceuticals for the treatment and prevention of COVID-19. We performed the search in the PubMed database and included variations of the search terms “SARS-CoV-2” or “coronavirus” or “COVID-19” AND “supplement” or “phytonutrient” or “nutraceutical” AND “review.” There were no restrictions on time period, language, or place of publication, and only review articles were included. This yielded 137 review articles after removing duplicates, from which titles and abstracts were reviewed. Sixty-seven articles were then removed for not pertaining to the research question and 25 for not being review articles, leaving 45 articles for full-text review and data extraction.

Our data extraction tool at this step focused on determining which nutraceuticals are most efficacious for the treatment or prevention of COVID-19 and included the name of the nutraceutical considered, the type of studies included in the review (e.g., in vitro, in vivo, animal or human studies), and the evidence for use against COVID-19.

For the purpose of our review, a nutraceutical was considered efficacious if our data extraction tool resulted in two or more reviews in favor of that nutraceutical’s ingestion for COVID-19, either through food or supplement form, and no reviews indicating harm from use. Nutraceuticals for which there was only one review in favor were searched again in PubMed for original articles. If this secondary search yielded two or more original results in its favor, that nutraceutical was also included. This process resulted in the inclusion of the following nutraceuticals for review of the safety of single-nutrient supplements in patients with cancer: vitamin D, vitamin C, zinc, selenium, omega-3 fatty acids, and quercetin (see Supplementary Materials).

At the next step of the process, for each of the identified “anti-COVID-19” nutrients, we conducted a new PubMed search regarding safety for use in patients with cancer. The search was performed using the nutraceutical name (e.g., “vitamin D”) AND “supplement” AND “cancer” AND “survivorship” or “safety” or “recurrence” or “disease progression” or “mortality” or “adverse events.” Additional articles were sourced from a hand-search of related literature by the included authors. After duplicate removal, this yielded 470 articles in total across all included nutraceuticals for review.

## 3. Results

**Figure 1:**
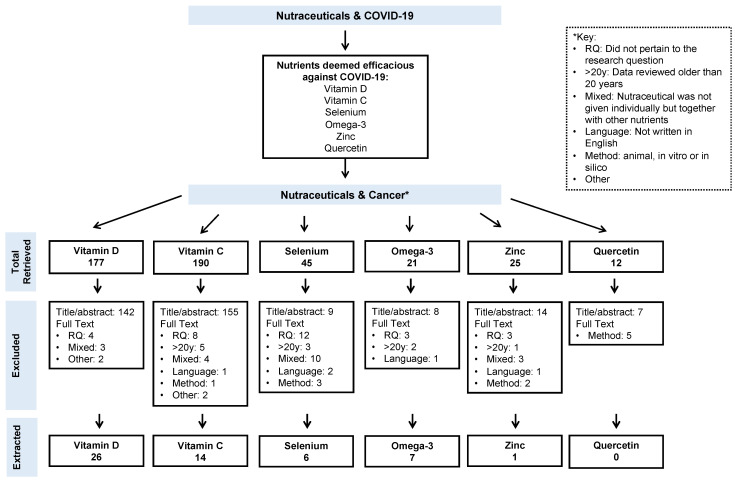
Search methodology and article selection process.

Out of 470 articles reviewed, 406 were excluded, leaving a total of 52 independent studies across all included nutraceuticals for data extraction (two of which included data for two nutraceuticals, resulting in a total of 54 records [35,36]). From those 52 studies, we extracted information about the authors, type of study, participants, cancer studied, nutraceutical dosing, and results. The search and selection process is graphically illustrated in Figure 1, and extracted information from the retrieved studies is shown in Table 1, Table 2, Table 3, Table 4 and Table 5.

### 3.1. Vitamin D

A total of 177 unique articles were retrieved for vitamin D through our PubMed search. We reviewed titles and abstracts, resulting in 35 for full-text review. After a full-text review, 26 articles remained for data extraction (Table 1).

Of those 26 studies, 23 reported results that indicated benefit, no harm, or null effects of vitamin D supplementation for patients with cancer. Two of the studies reported results with a negative impact for patients with cancer, and one study reported mixed results.

In the studies that found that vitamin D supplements were either beneficial or not harmful for patients with cancer, nine found that supplementation had no effect on a variety of outcomes including symptom management, risk of death, and risk of recurrence [43,51,52,54,57,58,59,60,61].

Three of the studies found that vitamin D was associated with better quality-of-life outcomes, including better scores on the cancer quality-of-life questionnaire (QLQ-C30) for physical functioning, social functioning, fatigue, and appetite, and better scores on the colorectal cancer subscale of the Functional Assessment of Cancer Therapy-Colorectal (FACT-C) tool [37,52,59]. Beyond quality-of-life measures, four studies reported a decrease in cancer mortality in those who took vitamin D supplements, and two showed a decrease in overall mortality [38,43,46,49,53,56]. One study found a lower risk of breast cancer recurrence in those who were supplemented with vitamin D post-diagnosis, but only among estrogen receptor (ER)-positive tumors and not among ER-negative tumors (HR = 0.64, 95% CI: 0.47–0.87 and HR = 1.25, 95% CI: 0.78–1.98; respectively) [35].

In the two studies that found vitamin D supplementation was harmful in patients with cancer, one found a positive association between vitamin D supplement use above 10 μg/day and cancer mortality (RR = 2.11, 95% CI: 1.18–3.77) [40], and the other found that vitamin D supplementation increased the risk of breast cancer mortality (HR = 1.47, 95% CI: 1.07–2.00) [48].

One RCT found mixed results for vitamin D supplementation with 200 IU/day in patients with digestive-tract cancer, post-curative surgery [55]. The study found that the effect of supplementation depended on the levels of serum Programmed Death Ligand 1 (PD-L1), a regulatory molecule expressed in T cells with immunosuppressive function [55]. Since PD-L1 is associated with a poorer cancer prognosis in various types of cancer (gastric cancer, small cell lung cancer, pancreatic cancer, breast cancer) [83,84,85,86], for those patients in the lowest PD-L1 concentration quintile, vitamin D supplementation seemed to have a detrimental effect by upregulating serum PD-L1 levels; however, for those in the highest quintile, vitamin D was beneficial and downregulated serum PD-L1 levels [55,87].

### 3.2. Vitamin C

A total of 190 unique articles were retrieved for vitamin C through our PubMed search. We reviewed titles and abstracts, resulting in 35 for full-text review. After a full-text review, 14 articles remained for data extraction (Table 2).

Of those 14 studies, all provided results in the direction of benefit, no harm, or null effects of vitamin C supplementation in patients with cancer. Six of the fourteen studies found no association between the use of vitamin C supplements and adverse cancer-related events, including recurrence, survival, overall mortality, and cancer-specific mortality [34,62,64,67,68,69]. Additionally, a study on chemotherapy-induced peripheral neuropathy found no significant effect of pre-treatment vitamin C supplementation on neurotoxicity [61]. Three studies found that vitamin C intake was associated with decreased overall mortality, three found a decreased risk of cancer-specific mortality, and three found a decreased risk of recurrence [35,63,65,66,70,71]. None of the studies reported an increased risk to health from the use of vitamin C supplements.

### 3.3. Selenium

A total of 45 unique articles were retrieved for selenium through our PubMed search. We reviewed titles and abstracts, resulting in 28 for full-text review. After a full-text review, six articles remained for data extraction (Table 3).

Five of these six papers showed no harmful effects of selenium supplementation in patients with cancer and included two meta-analyses [72,73], two reviews [62,76], and one RCT [75]. Three articles did not find a beneficial effect on the incidence or progression of gastrointestinal cancer [62], prostate cancer [73], or cervical and uterine cancer [75], but found selenium supplementation was not otherwise harmful. Beneficial effects were highlighted in a review that addressed an association between selenium supplementation and decreased edema volumes and incidence of skin infection in patients with breast cancer in an RCT of 179 post-mastectomy patients with secondary lymphedema, as well as decreased edema volumes in 10 out of 12 patients with breast cancer included in a 48-participant cohort study [76].

A meta-analysis of RCTs by Jenkins et al. concluded that selenium taken independently (i.e., not as a multivitamin or mixed with other supplements) was not associated with cancer mortality [72]. However, a prospective cohort study within the review found that high-dose selenium supplementation (≥140 μg/day) may be associated with a greater risk of prostate cancer mortality [72].

### 3.4. Omega-3 Fatty Acids

A total of 21 unique articles were retrieved for omega-3 fatty acids through our search. We reviewed titles and abstracts, resulting in 17 for full-text review. After a full-text review, seven articles remained for data extraction (Table 4).

In five of these seven studies, there were no adverse effects of supplementation. One study found that supplementation with omega-3 fatty acids decreased aromatase-inhibitor-related pain in patients with breast cancer and obesity [79]. Additionally, omega-3 supplementation showed promising antitumor activity in two prospective trials of patients with advanced lung and breast cancer, as reviewed by Vernieri et al. [81]. The same review, however, highlighted a pre-clinical study that reported that the 16:4 omega-3 (hexadeca-4,7,10,13-tetraenoic) fatty acid supplement, commonly found in commercial fish oils, may be unsafe for patients with cancer as it can hinder tumor-directed cytotoxicity of platinum compounds used in cancer treatments [81].

Furthermore, an RCT pointed towards an increased mortality rate 5 years after patients with colorectal cancer (from a country with traditionally high fish intake) took omega-3 supplements in the 7 days before and after colorectal resection surgery [80].

### 3.5. Zinc

A total of 25 unique articles were retrieved for zinc through our PubMed search. We reviewed titles and abstracts, resulting in 11 for full-text review. After a full-text review, only one article remained for data extraction (Table 5).

The study found that zinc supplementation reduced the duration and severity of oral mucositis in patients with head and neck cancer but sometimes caused gastrointestinal distress, which suggests that zinc supplements should not be taken on an empty stomach [88].

### 3.6. Quercetin

A total of 12 unique articles were retrieved for quercetin through our PubMed search. We reviewed titles and abstracts, resulting in five for full-text review. After a full-text review, one was removed for being conducted in animals, and the remaining four were review articles that did not include human studies; therefore, no articles qualified for further consideration.

## 4. Discussion

This narrative review aimed to synthesize the currently available literature regarding the safety of the most efficacious “anti-COVID-19” nutraceuticals for patients with cancer. Our findings reveal heterogeneous results, with safety largely depending on the type of nutraceutical or supplement consumed, the dose consumed, and the type of cancer studied. Across nutraceuticals, our results were heavily based on observational studies. Taking the potential risk of confounding into consideration, clear conclusions could not be drawn, further emphasizing the need for caution from healthcare providers.

Vitamin D may decrease CRP, which has been implicated in the cytokine storm seen in severe cases of COVID-19 infection [18]. We identified an overwhelming majority of studies with results that point in favor of vitamin D use in patients with cancer, with positive effects seen in quality-of-life measures, mortality, recurrence, and pain indexes. However, the mechanism between vitamin D and these positive cancer-related outcomes was not always well characterized. Anderson et al. documented improved quality-of-life measures in an observational cohort of patients with breast cancer, but ultimately noted that it was unclear whether the supplement itself was responsible or whether participants who took vitamin D were in general more optimistic or more likely to take other actions towards improving their overall health and mood [37]. Similarly, Bjelakovic et al. reported decreased cancer mortality from vitamin D3 supplementation (RR = 0.88, 95% CI: 0.78–0.98) but noted the lack of RCTs made it hard to draw robust conclusions [38].

Only two studies point to an increased risk of vitamin D intake in patients with cancer; one noted this was observed only among those who were not deficient in vitamin D [40], and the other noted that the association of vitamin D supplementation with higher breast cancer mortality needed further exploration, as there was no clear mechanism behind this observation [48]. Given that the majority of evidence is in support of vitamin D use, oncologists can likely safely allow their patients to continue supplementation at typically recommended doses (600 IU/day).

Vitamin C, similar to vitamin D, may contribute to a decrease in the pro-inflammatory cytokines, which are a hallmark of severe COVID-19 infection [18]. The evidence for vitamin C also strongly points in the direction of supplementation being safe, or perhaps even beneficial, for patients with cancer. In fact, none of the included articles found an indication of harm. Given that there has been concern that the use of antioxidants, including vitamin C, may negatively impact the effect of chemotherapeutic agents, these results are encouraging [70]. Nevertheless, we urge caution as the studies are, by and large, observational in nature, which stresses the need for additional clinical trials [49]. At the present state of knowledge, supplementation with vitamin C at typically recommended doses (75–90 mg/day) is likely not harmful and could conceivably confer benefit.

Selenium may reduce the severity of COVID-19 infection by impeding viral entry into the cytoplasm and has promising results in patients with cancer [89]. All but one out of six studies addressing selenium supplementation demonstrated no adverse effects in patients with cancer. However, the type of cancer (i.e., prostate, uterine, cervical, gastrointestinal) and outcome of interest varied greatly across studies. One prospective cohort study in patients with prostate cancer noted that selenium supplementation might be associated with a higher risk of mortality if intake is high (≥140 μg/day) [72]. Given these results, it is likely that selenium use is safe for patients with cancer, though high-dose supplementation should be avoided (typically recommended doses: 40–70 mg/day).

Omega-3 fatty acids may play a role in decreasing the severity of COVID-19 infection by inhibiting cellular viral entry, suppressing the production of pro-inflammatory cytokines, and increasing the phagocytic capacity of the innate immune system [20]. Out of seven identified articles for omega-3 fatty acids, five found their use to be safe, though estimates of efficacy varied [39,77]. The seven articles addressed safety in a variety of different cancers, including skin cancer, prostate cancer, gastrointestinal cancer, breast cancer, esophageal cancer, glioblastoma, and colorectal cancer. Two studies evaluated the long-term effects of supplementation with omega-3 fatty acids. The first, a longitudinal cohort study, did not find an association between mortality and supplementation in glioblastoma patients [56]. In contrast, the other, an RCT, pointed towards an increased mortality rate after five years of intake in patients with colorectal cancer who supplemented one week before and one week after colorectal resection surgery [80]. Additionally, one review specifically warned against the indiscriminate use of fish oil supplements, which may be unsafe for patients with cancer if they contain hexadeca-4,7,10,13-tetraenoic acid; this omega-3 fatty acid can dampen the tumor-directed cytotoxicity of platinum compounds used to treat some cancers [81]. Based on this evidence, caution should be used as far as omega-3 fatty acid supplements are concerned. At the very least, scrutiny of the exact fatty acid composition of the supplement together with frequent patient monitoring is warranted.

For zinc, which may counteract inflammation associated with tumor necrosis factor-α in COVID-19 infection [90], results did not universally show harm-free supplementation. Although one study indicated a reduced incidence of oral mucositis with supplementation in patients with head and neck cancer, the same study also cited potential gastrointestinal distress at the same dosage [88]. Given the lack of a sufficiently large body of evidence on this nutraceutical, with only one study being relevant, it is hard to draw any conclusions. That said, at present, it is probably prudent to advise patients with cancer against supplementation with zinc.

While this review thoroughly and systematically assessed the literature regarding the safety of these supplements for patients with cancer, our conclusions are not without limitations. The heterogeneity of results may in part be due to our inclusion of all stages and types of cancer, as well as our inclusion of all treatment types and clinical settings. It is possible that a narrower scope would have revealed more homogenous results due to the vast differences in the biology of various cancers. However, at the current state of knowledge, there is not enough information for a cancer type-specific assessment. Additionally, our review did not consider in detail possible toxicity issues resulting from supra-supplementation but rather evaluated safety at typically recommended doses. Lastly, due to the relatively recent onset of the COVID-19 pandemic, there are limited clinical trials on the efficacy of nutraceuticals for SARS-CoV-2. As a result, data on only a limited number of nutraceuticals could be identified. As more research becomes available, it is possible that more nutraceuticals will be deemed efficacious, and an updated safety review may become necessary.

## 5. Conclusions

Patients with cancer are one of several co-morbid populations who are at increased risk of a severe course of disease if infected with COVID-19. While a number of nutraceuticals have attracted interest due to their potential “anti-COVID-19” activity, there is concern about the safety of their usage in patients with cancer due to the potential interactions with their treatment regimen and possible associations with an increased risk of recurrence, cancer incidence, or even death.

This review highlights the heterogeneity of results regarding the safety of nutraceuticals for patients with cancer. It is conceivable that a large part of this heterogeneity is due to different types and stages of cancer, different treatments, and different clinical settings among the identified studies. Our findings indicate that vitamin D, vitamin C, and selenium supplementation are likely safe at normal doses (i.e., the dosages typically recommended for the general population). However, caution should be used with omega-3 fatty acid supplementation due to a conflict in the results between two long-term studies and a paucity of data overall. Similarly, zinc supplementation should probably be avoided due to a lack of relevant studies and because the currently available evidence indicates potential for harm or discomfort in patients with cancer.

Overall, this work emphasizes a sizeable gap in the literature surrounding the safety of nutraceuticals in patients with cancer and underscores the potential danger of liberal use of supplements by this high-risk group. Furthermore, this review provides important and immediately relevant clinical guidance for cancer care practitioners during an ongoing public health crisis. It is important to note that any supplement intake by patients with cancer should be discussed with their healthcare team so their providers may more accurately monitor their health and assess potential risks. Lastly, though early evidence indicates a potential benefit of some nutraceuticals against COVID-19, and thus potentially to high-risk cancer populations, we do not recommend supplementation as a substitute for regular medical care and a balanced diet.

## Figures and Tables

**Table 1:** Safety of vitamin D supplements for patients with cancer.

Study	Type	Participants	Cancer	Dosage	Outcomes	Safety
Andersen et al., 2019 [37]	Observational	*n* = 553 patients with breast cancer/survivors (193 from cohort saw naturopathic physicians specializing in oncology, 360 usual care cohort) Age (mean ± SD) oncology cohort 53 ± 11 y; usual care cohort 55 ± 10 y Female BMI ^1^ not reported	Breast cancer All stages Therapy: chemotherapy and/or radiation	>50% reported taking <1000 IU daily	Users reported ↑ physical function, role-physical function, social function, and role-emotional function on the SF-36 HRQOL ^2^ assessment subscales at baseline (*p* < 0.05) At 6-month follow-up, users at baseline reported ↑ role-physical function, less pain, better general health, and ↑ vitality and social function (*p* < 0.05) Users at 6-month follow-up reported ↑ social function and mental health when assessed at the 12-month follow-up (*p* < 0.05)	(+)
Bjelakovic et al., 2014 [38]	Cochrane review	*n* = 50,623 Age (range) 18–107 y Male and female BMI not reported	All cancers All stages Therapy not specified	Not reported	Users had ↓ cancer mortality (RR = 0.88, 0.78–0.98, *p* = 0.02; 44,492 participants; 4 trials)	(+)
Campbell et al., 2021 [39]	Intervention	*n* = 68 Age (range) 59–67 y Male BMI not reported	Prostate cancer Stage 1 Therapy not specified	Dose titrated to achieve serum levels of 60 ng/mL Administered periodically	Participants with ↑ initial vitamin D levels were twice as likely to have ↓ prostate-specific antigen slope (OR = 2.04, 1.04–4.01, *p* = 0.04)	(+)
Chen et al., 2019 [40]	Prospective cohort study	*n* = 30,899 Age 20+ y Male and female *n* of non-users/users per BMI category, 4301/4401 (<25 kg/m^2^), 5119/4862 (25–30 kg/m^2^), 5483/4388 (≥30 kg/m^2^)	All cancers All stages Therapy not specified	Evaluated use as >10 mg/d from a 30-day questionnaire	Users had ↑ risk of cancer mortality (RR = 2.11, 1.18–3.77)	(−)
Chlebowski et al., 2013 [41]	Literature review	*n* ranged from 200 to >100 participants per study Age not reported Male and female BMI not reported	Breast cancer All stages Therapy: bisphosphonate, chemotherapy, aromatase inhibitor therapy, letrozole, zoledronic, or unspecified	Varied based on study	Prospective cohort studies showed no association between ↑ 25(OH)D ^3^ levels and ↓ breast cancer incidence Studies of vitamin D and subsequent breast cancer recurrence were mixed ↓ vitamin D levels associated with ↑ risk of recurrence in analyses not controlled for prognostic variables, cancer therapy, BMI, and physical activity ↑ prevalence of ↓ vitamin D levels seen in early-stage breast cancer, but control population information is lacking 1 RCT ^4^ did not demonstrate ↓ breast cancer incidence in postmenopausal women (1000 mg of calcium and 400 IU vitamin D3 daily in intervention group compared to placebo)	(+)
Chowdhury et al., 2014 [42]	Systematic review and meta-analysis	*n* = 849,412 in observational studies *n* = 30,716 in interventional Age not reported Male and female BMI not reported	All cancers All stages Therapy not specified	Varied based on study	Observational studies report associations of ↓ circulating 25(OH)D with ↑ risk of mortality from cancer	(+)
Cook et al., 2010 [43]	Meta-analysis	Total *n* not reported Age not reported Sex not reported BMI not reported	Ovarian cancer Stage not reported Therapy not specified	Varied based on study	About half of the case-control studies reported ↓ mortality with ↑ latitude, solar radiation, or dietary intake or supplementation, and the rest had null associations Cohort studies found no risk reduction with ↑ dietary intake or supplementation pre-diagnosis (note: vitamin D intakes were low in all studies)	(+)
Datta et al., 2012 [44]	Review	Total *n* not reported Age not reported Sex not reported BMI not reported	Prostate cancer All stages Therapy: androgen deprivation therapy	Varied based on study	Clinical trial evidence does not show that supplementation with calcium and vitamin D prevents loss of bone mineral density during androgen deprivation therapy	(+)
Du et al., 2017 [45]	Review	Total *n* not reported Age not reported Sex not reported BMI not reported	Gastric cancer All stages Therapy not specified	Varied based on study	Inconsistent results on efficacy Vitamin D deficiency may ↑ the risk and mortality of gastric cancer	(+)
Grant et al., 2019 [46]	Review	Total *n* not reported Age not reported Sex not reported BMI not reported	All cancers All stages Therapy not specified	Varied based on study	Meta-analysis of 10 RCTs involving 45,197 participants found vitamin D use (variable dose and duration) was associated with 15% ↓ cancer mortality (RR = 0.85, 0.75–0.96) Vitamin D deficiency may ↑ risk and mortality of gastric cancer 1 RCT found women with a serum 25(OH)D concentration >40 ng/mL had 65% ↓ all-cancer incidence rate than women with values <20 ng/mL	(+)
Harvie et al., 2014 [47]	Review	Total *n* not reported Age not reported Sex not reported BMI not reported	Prostate, hematologic cancers, melanoma, breast, colorectal, lung cancers All stages Therapy: 1 RCT in prostate cancer included docetaxel chemotherapy; therapy not reported in other trials	Not reported	1 RCT showed positive results (longer survival time) in patients with advanced prostate cancer receiving docetaxel chemotherapy	(+)
Holm et al., 2014 [48]	Prospective cohort	*n* = 1064 Age not reported Female BMI (median) 24.7 kg/m^2^	Breast cancer Stage not reported Therapy: hormone replacement therapy vs. no therapy pre-diagnosis	Not reported	Use was associated with ↑ breast cancer mortality (HR = 1.47, 1.07–2.00)	(−)
Kanellopoulou et al., 2021 [49]	Meta-analysis	Total *n* not reported Age not reported Sex not reported BMI not reported	All cancers All stages Therapy not specified	Not reported	In breast cancer survivors, use ↓ risk of total mortality (RR = 0.85, 0.72–0.99)	(+)
Khan et al., 2017 [50]	RCT	*n* = 160 Age (range) 54–69 y Female Average group BMI (placebo/supplementation) was 29.6/29.9 kg/m^2^, respectively	Breast cancer All stages Therapy: chemotherapy and/or radiation	30,000 IU vitamin D3 weekly	Scores for measures of pain intensity in BPI ^5^ were better in women randomized to vitamin D compared to placebo Worsening of aromatase inhibitor-associated musculoskeletal symptoms observed in 71% of subjects randomized to placebo (plus the standard supplement of 600 IU of D3/day) vs. 40% of subjects randomized to high dose vitamin D3 plus the standard supplemental dose (*p* < 0.001) Six months of oral vitamin D3 at 30,000 IU/week was safe in women starting an aromatase inhibitor for adjuvant treatment of breast cancer and is effective to ↑ serum 25(OH)D levels	(+)
Klapdor et al., 2012 [51]	Prospective cohort	*n* = 248 ambulatory patients (*n* = 103 with pancreatic cancer) Age not reported Sex not reported BMI not reported	Pancreatic cancer Stage not reported Therapy: pancreatic enzyme drugs	Vitamin D oral to ↑ serum levels to >30 ng/mL in group II and in the patients of group III in order to reach stable serum 25(OH)D concentrations in the normal range Doses varied	Oral vitamin D can be supplied without side-effects	(+)
Lewis et al., 2016 [52]	Prospective cohort	*n* = 453 Age (mean) 63.3 y Male and female BMI (mean) 28.7 kg/m^2^	Colorectal cancer Stage II Therapy: any	Not reported	No association between vitamin D use and risk of recurrence or mortality Beneficial association between use and functional assessment in colorectal cancer subscale of the FACT-C ^6^ (*p* = 0.04)	(+)
Madden et al., 2018 [53]	Longitudinal cohort	*n* = 5417 Age at diagnosis (range) 50–80 y Female BMI not reported	Breast cancer Stage I–III Therapy: any	Categories of no use, 1–400 IU/day, and >400 IU/day	20% ↓ in breast cancer-specific mortality in de novo vitamin D users vs. non-users (HR = 0.80, *p* = 0.048) 49% ↓ breast cancer-specific mortality if vitamin D initiated within 6 months of breast cancer diagnosis (HR = 0.51, *p* < 0.001)	(+)
Martinez et al., 2012 [54]	Review	Total *n* not reported Age not reported Sex not reported BMI not reported	All cancers Stage not reported Therapy not specified	Not reported	One RCT showed no effect of use on cancer mortality One RCT showed no effect of use in breast or colorectal cancer incidence with vitamin D plus calcium One RCT showed ↓ in total cancer incidence with vitamin D plus calcium vs. placebo	(+)
Morita et al., 2021 [55]	Post-hoc analysis of RCT	*n* = 396 Age (median) 66 y Male and female BMI (median) 21.9 kg/m^2^	Digestive tract Stage I–III Therapy: post- curative surgery with complete tumor resection	200 IU/day vs. placebo, until relapse or death	In lowest PD-L1 ^7^ quintile, vitamin D upregulated serum PD-L1 levels (*p* = 0.0008); no change with placebo In the highest quintile, vitamin D downregulated serum PD-L1 levels (*p* = 0.0001); no change with placebo A significant effect of vitamin D on death, compared with placebo, only in the highest PD-L1 quintile (HR = 0.34, 0.12–0.92); not observed in other quintiles Significant effect of vitamin D on death or relapse, compared with placebo, only in the highest PD-L1 quintile (HR = 0.37, 0.15–0.89)	(+/−)
Mulpur et al., 2015 [56]	Cohort	*n* = 470 Age (median) 59 y Male and female BMI not reported	Glioblastoma High grade Therapy: standard of care treatment involving surgery, chemotherapy, and radiation therapy	Not reported	Vitamin D use associated with ↓ age-adjusted mortality (HR = 0.68, *p* = 0.019) and after multivariate adjustment (HR = 0.72, *p* = 0.043) Results for vitamin D attenuated when the reference category confined to non-alternative medicine users in a multivariate model	(+)
Poole et al., 2013 [35]	Cohort	*n* = 12,019 Age (mean) 56.8 y Female Frequency of BMI < 25 kg/m^2^, 25–30 kg/m^2^, and ≥30 kg/m^2^ was roughly 50%, 30%, and 20%, respectively	Breast cancer Excluded in situ or stage IV Therapy: varied—chemotherapy, radiation, or hormone therapy present in cohort	Not reported	Vitamin D use was associated with ↓ risk of recurrence in ER+ ^8^ (HR = 0.64) but not in ER– tumors (HR = 1.25) Stratified by joint ER/PR status, vitamin D was only associated with ↓ risk of recurrence in ER+/PR+ ^9^ and ER+/PR− tumors, but not ER−/PR+ or ER−/PR− tumors (*p* = 0.002 for interaction)	(+)
Saquib et al., 2011 [57]	Cohort derived from RCT	*n* = 3081 Age (mean) 53 y Female 24% of users and 36% of non-users had obesity	Breast cancer Operable invasive stage I (≥1 cm), II, or IIIA Therapy: none (study done in survivors)	6 μg/day total intake of vitamin D in those who took supplements	No significant findings related to all-cause mortality	(+)
Sarre et al., 2016 [58]	Cohort from men participating in the third round of the FinRSPC ^10^ randomized screening study	*n* = 12,740 Ages: 63, 67, or 71 y Males BMI not reported	Prostate cancer Stage not reported Therapy not specified	Not reported	Vitamin D use had no association with prostate cancer incidence, high-grade/metastatic cancers, or death	(+)
Wang et al., 2016 [59]	Longitudinal observational	*n* = 303 Age of users and non-users (means) 62 and 65 y, respectively Predominately male BMI (mean) 21 kg/m^2^	Esophageal cancer Roughly 65% stage 0/I/II, 35% stage III/IV, 44% with lymph node involvement Therapy: esophagectomy and some with postoperative chemotherapy and/or radiotherapy	200–400 IU/day for 1 year	Associations between use and QOL ^11^, including global health, physical functioning, social functioning, fatigue, and appetite loss measured by QLQ-C30 ^12^ Users more likely to have improved disease-free survival (*p* = 0.030) No association of use with overall survival	(+)
Zhang et al., 2019 [60]	Meta-analysis of RCTs	*n* = 77,653 from 9 studies Age (range) 20–84 y Male and female BMI not reported	All cancers Staging not reported Therapy not specified	Varied across 9 studies	No significant effect on cancer incidence or mortality	(+)
Zirpoli et al., 2017 [61]	Cohort	*n* = 922 Age not reported Female BMI not reported	Breast cancer Stage I–III breast cancer (node-positive (pN1–3) Any primary tumor ≥ 2 cm, or any tumor ≥ 1 cm if estrogen receptor negative/progesterone receptor negative or hormone receptor positive with 21-gene recurrence score ≥26 Therapy: paclitaxel (1/week for 12 weeks or every other week)	Not reported	No improvement in peripheral neuropathy Fact-NTX ^13^ or CTCAE ^14^ scores	(+)

Abbreviations used: ^1^ Body Mass Index, ^2^ Short Form Health-Related Quality of Life, ^3^ 25-hydroxy vitamin D, ^4^ Randomized Controlled Trial, ^5^ Brief Pain Index, ^6^ Functional Assessment of Cancer Therapy—Colorectal, ^7^ Programed death ligand 1, ^8^ Estrogen Receptor, ^9^ Progesterone Receptor, ^10^ Finnish Randomized Study for Screening of Prostate Cancer, ^11^ Quality of Life, ^12^ Quality of Life Questionnaire-Core Questionnaire, ^13^ Functional Assessment of Cancer Therapy-Neurotoxicity, ^14^ Common Terminology Criteria for Adverse Events. The last column indicates the overall direction of the effects of vitamin D supplementation on safety: (+) no risks to health; (−) some risks to health outcomes; (+/−) mixed risk profile. Relative risks (RR) and odds/hazard ratios (OR/HR) are shown as means with 95% confidence intervals.

**Table 2:** Safety of vitamin C supplements for patients with cancer.

Study	Type	Participants	Cancer	Dosage	Outcomes	Safety
Ambrosone et al., 2020 [34]	Correlative analysis from SWOG S0221	*n* = 1134 Age (mean) progression free 50.9 y Age (mean) with progression 52.8 y Female BMI ^1^ (mean) progression free 29.1 kg/m^2^ BMI (mean) with progression 30.1 kg/m^2^	Breast cancer Stage not available, most node negative Randomized to treatment of cyclophosphamide, doxorubicin, and paclitaxel	Not reported	No association with use of vitamin C before and during treatment and recurrence (HR = 1.36, 0.87–2.13) No association with vitamin C and overall survival	(+)
Bjelakovic et al., 2008 [62]	Systematic review	*n* not reported Age not reported Male and female BMI not reported	Gastrointestinal cancer Stage not reported Therapy not specified	Dose ranged 120–2000 mg/day depending on the trial	Vitamin C supplement use (RR = 0.97, 0.77–1.23) did not influence mortality Combination vitamin C with beta-carotene, vitamin E, and selenium did not influence mortality compared to placebo	(+)
Greenlee et al., 2012 [63]	Cohort	*n* = 2264 Age (range) 18–79 y Female BMI not reported Majority had BMI < 25 kg/m^2^	Breast cancer Stage I–IIIA Therapy completed	Categories of no use, occasional use (<1–5 days/week), and frequent use (6–7 days/week) No details on dose	Frequent use of vitamin C associated with ↓ risk of breast cancer recurrence (HR = 0.73, 0.55–0.97)	(+)
Harris et al., 2013 [64]	Cohort	*n* = 3405 Age (mean) at dx ^2^ = 65 y Female Mean BMI = 25 kg/m^2^	Breast cancer All stages All therapies	≈1000 mg/day	No association between vitamin C supplement use and breast cancer-specific mortality (HR = 1.06, 0.52–2.17).	(+)
Harris et al., 2014 [65]	Meta-analysis	*n* not reported Age not reported Female BMI not reported	Breast cancer Stage not reported All therapies	Various	Post-diagnosis usage reduced breast cancer-specific mortality (RR = 0.85, 0.74–0.99)	(+)
Jacobs et al., 2002 [66]	Cohort	*n* = 942,993 Age 30+ y Male and female BMI not reported	Stomach cancer Stage not reported Therapy not specified	Not reported	Regular vitamin C use tended to ↓ risk of stomach cancer mortality (RR = 0.83, 0.68–1.01) ↓ risk only in participants using vitamin C for a relatively short duration of time (RR = 0.68, 0.51–0.91 for <10 years use; RR = 1.00 0.73–1.38 for ≥10 years use)	(+)
Jacobs et al., 2002 [67]	Cohort	*n* = 991,552 Age not reported Male and female BMI not reported	Bladder cancer Stage not reported All therapies	Not reported	Regular vitamin C supplement use (≥15 times per month) not associated with bladder cancer mortality	(+)
Kanellopoulo et al., 2020 [49]	Meta-analysis	*n* not reported Age 18+ y Male and female BMI not reported	All cancers Stage 0–IV All therapies	Not reported	In breast cancer survivors, vitamin C supplement use associated with ↓ total mortality Vitamin C supplement use associated with ↓ breast cancer recurrence (RR = 0.76)	(+)
Lin et al., 2009 [68]	RCT ^3^	*n* = 7627 Age (mean) 60.4 y Female BMI (mean) 30 kg/m^2^ in Vitamin C group	Any cancer No dx at baseline Therapy: none	500 mg/day	No effects of use of any antioxidant on cancer incidence. Vitamin C vs. placebo, no difference in mortality	(+)
Messerer et al., 2008 [69]	Cohort	*n* = 38,994 Age (range) 45–79 y Male BMI not reported	All cancers No cancer at baseline Therapy: none	Estimated 1000 mg/day	No association between use of any dietary supplementation and all-cause mortality, cancer, or CVD ^4^ mortality	(+)
Nechuta et al., 2011 [70]	Cohort	*n* = 4877 Age (range) 20–75 y Female BMI not reported	Breast cancer Stage I–IV All therapies	Majority consumed < 400 mg/day supplement	Use of vitamin C for >3 months had a 44% ↓ in risk of mortality and 38% ↓ in risk of recurrence	(+)
Pocobelli et al., 2009 [71]	Cohort	*n* = 77,719 Age 50–76 y Male and female BMI not reported	All cancers All stages Therapy not specified	Varied	Vitamin C use associated with ↓ risk of cancer mortality, but no dose–response trend	(+)
Poole et al., 2013 [35]	Cohort	*n* = 12,019 Age (mean) 56.8 y Female Frequency of BMI was roughly 50% <25 kg/m^2^, 30% 25–29.9 kg/m^2^, 20% above 30 kg/m^2^	Breast cancer Excluded in situ or stage IV Therapy: varied—chemotherapy, radiation, or hormone therapy	Not reported	Vitamin C use associated with ↓ risk of death (RR = 0.81) Use of antioxidant supplements (multivitamins, vitamin C or E) not associated with recurrence	(+)
Zirpoli et al., 2017 [61]	Cohort	*n* = 922 Age not reported Female BMI not reported	Breast cancer Stage I–III (node-positive (pN1–3) Any primary tumor ≥ 2 cm, or any tumor ≥ 1 cm estrogen receptor negative/progesterone receptor negative or hormone receptor positive with 21-gene recurrence score ≥ 26) Therapy-Paclitaxel (1x/week for 12 weeks or every other week)	Not reported	Use of vitamin C, folic acid, calcium, iron, or fish oil before diagnosis was not associated with CTCAE ^5^ grade 3 or 4 neurotoxicity	(+)

Abbreviations used: ^1^ Body Mass Index, ^2^ diagnosis, ^3^ Randomized Control Trial, ^4^ Cardiovascular disease, ^5^ Common Terminology Criteria for Adverse Events. The last column indicates the overall direction of the effects of vitamin C supplementation on safety: (+) no risks to health; (−) some risks to health outcomes; (+/−) mixed risk profile. Relative risks (RR) and odds/hazard ratios (OR/HR) are shown as means with 95% confidence intervals.

**Table 3:** Safety of selenium supplements for patients with cancer.

Study	Type	Participants	Cancer	Dosage	Outcomes	Safety
Bjelakovic et al., 2008 [62]	Systematic review of RCTs ^1^	*n* = 211,818 participants total in 20 RCTs Age (mean) 56.5 y (range 15–84 y) Male (59%) and female BMI ^2^ not reported	Gastrointestinal cancer All stages Therapy not specified	Not reported	Selenium use (singly or with other antioxidants) significantly ↓ mortality (RR = 0.90, 0.83–0.98), effect attenuated when high-risk trials excluded	(+)
Jenkins et al., 2020 [72]	Systematic review/meta-analysis of RCTs	*n* not reported Age not reported Male and female BMI not reported	All cancers All stages (and mortality) Therapy not specified	Not reported	Selenium supplement use, singly or with other antioxidants, was not associated with cancer incidence or cancer mortality	(+)
Jiang L et al., 2010 [73]	Meta-analysis of RCTs	*n* = 165,056 participants across 9 RCTs Age not reported Male BMI not reported	Prostate cancer All stages Therapy not specified	Not reported	Mortality among patients with prostate cancer did not significantly differ by selenium supplementation (RR = 2.98, 0.12–73.2) Incidence/mortality of prostate cancer did not ↓ with selenium supplement intake	(+)
Kenfield et al., 2015 [74]	Prospective cohort study	*n* = 4459 Age (mean) 68.9 +/− 7.2 y at diagnosis Male BMI (mean) 25.8 kg/m^2^	Prostate cancer Not metastatic at diagnosis Therapy: radical prostatectomy, EBRT ^3^ or brachytherapy, hormones, watchful waiting, or other	1–24 μg/day, 25–139 μg/day or 140+ μg/day of selenium supplement	No ↑ risk of prostate cancer mortality in 1–24 μg/day and 25–139 μg/day selenium supplementation ↑ risk of prostate cancer mortality in 140+ μg/day selenium supplementation (RR = 2.60, 1.44–4.70) vs. those not taking supplement	(+/-)
Muecke R et al., 2010 [75]	RCT	*n* = 81 Age (mean) 64.3 ± 10.1 y; (range) 31–80 Female BMI not reported	Cervical and uterine cancer All stages Therapy: radiation therapy	Radiation therapy days = 500 μg of selenium Other days = 300 μg of selenium 17 mg of sodium selenite given cumulatively over average treatment period of 38 days	In 10 years of follow-up, no difference in disease-free survival between selenium group and control (*p* = 0.65) No difference in 10-year overall survival rate in selenium group vs. control (*p* = 0.09)	(+)
Samuels et al., 2014 [76]	Review	Total *n* not reported Age not reported Sex not reported BMI not reported	Breast cancer All stages Therapy in 1 RCT: standard combined decongestion therapy	1 RCT—1st week = 1000 μg/d, 2nd week = 300 μg/d, final weeks = 100 μg/d for 3 total months 1 cohort = 350 μg/m^2^ daily for 4–6 weeks	1 RCT: 179 post-mastectomy patients with secondary lymphoedema. Selenium supplement use ↓ in edema volumes incidence of skin infections vs. controls 1 cohort: 48 patients with post-radiation lymphoedema (12 patients also had breast cancer). 83.3% of those with cancer had ↓ in edema with supplementation	(+)

Abbreviations used: ^1^ Randomized Controlled Trial, ^2^ Body Mass Index, ^3^ External Beam Radiation Therapy. The last column indicates the overall direction of the effects of selenium supplementation on safety: (+) no risks to health; (−) some risks to health outcomes; (+/−) mixed risk profile. Relative risks (RR) and odds/hazard ratios (OR/HR) are shown as means with 95% confidence intervals.

**Table 4:** Safety of omega-3 fatty acid supplements for patients with cancer.

Study	Type	Participants	Cancer	Dosage	Outcomes	Safety
Campbell et al., 2021 [39]	Intervention	*n* = 68 Age (range) 59.3–66.9 y Male BMI ^1^ not reported	Prostate cancer Stage 1 (very low or low risk) Therapy not specified	720 mg (3/day)	Relationship between prostate-specific antigen slope and initial total omega-3 levels were not statistically significant (r = 0.05; *p* = 0.792) Similarly not significant for initial omega-6:3 ratio (r = −0.1; *p* = 0.95), final omega-3 levels (r = 0.16; *p* = 0.531), and final omega-6:3 ratio (r = −0.28; *p* = 0.282) Study cohort had no pathologic or clinical progression and no serious side effects from omega-3 supplement use	(+)
Klassen et al., 2020 [77]	Review article	*n* = 140 participants across studies Age not reported Male and female BMI not reported	Breast and gastrointestinal cancers All stages Therapy: chemotherapy or otherwise not specified	Varied across studies	All study results support safety/tolerability of omega-3 supplement during chemotherapy Evidence supporting benefits for omega-3 supplement in breast and gastrointestinal cancer is weak	(+)
Miyata et al., 2017 [78]	RCT ^2^	*n* = 61 patients Age (range) 56.1–72.7 y 52 male, 9 female BMI: Omega-3 group (mean) 21.8 +/− 10 kg/m^2^, placebo group (mean) 20.8 +/− 7.1 kg/m^2^	Esophageal cancer All stages Therapy: neoadjuvant chemotherapy	900 mg/day omega-3 in intervention group and 250 mg/day in comparison group Both groups had enteral nutrition supplement provided 3 days before initiation of chemotherapy to day 12 of chemotherapy	No difference in incidence of grade 3/4 neutropenia between both groups (77.4% in intervention vs. 83.3% in comparison *p* = 0.561) or frequency (93.5 in intervention vs. 86% in comparison, *p* = 0.363) Omega-3 enteral nutrition support ↓ frequency of chemotherapy-induced mucosal toxicities and prevented increase in the aspartate amino transferase and alanine amino transferase levels	(+)
Mulpur et al., 2015 [56]	Longitudinal cohort	*n* = 106 Age (range) 18–84 y Male and female BMI not reported	Glioblastoma All stages Therapy: surgery, chemotherapy, radiation	Not reported	No effect of omega-3 supplementation on mortality	(+)
Shen et al., 2018 [79]	Exploratory analysis of RCT	*n* = 249 Age (median) 59 y Females 56% = BMI < 30 44% = BMI ≥ 30	Breast cancer Stages I–III Aromatase-inhibitor therapy	3.3 g/day (560 mg EPA ^3^ plus DHA ^4^ acid in a 40:20 ratio) omega-3 in intervention group and placebo (soybean-corn oil blend) in comparison group for 24 weeks	Omega-3 supplement use associated with ↓ BPI ^5^ worst pain scores vs. placebo (4.36 vs. 5.70, *p* = 0.02) in patients with obesity No difference in scores between treatment arms (5.27 vs. 4.58, *p* = 0.28; *p* = 0.05) in patients who weren’t obese Omega-3 supplement use in patients with obesity was associated with ↓ BPI average pain and pain interference scores vs. placebo (*p* = 0.005)	(+)
Sorensen et al., 2020 [80]	RCT	*n* = 148 Age (mean) 68.3 +/− 11.3 y Males and female BMI not reported	Colorectal cancer All stages Therapy: surgery	Intervention group, 2.0 g EPA and 1.0 g DHA per day No EPA/DHA for control group	No difference in 5-year survival for intervention group vs. control (*p* = 0.193) Adjusted for age/disease stage/therapy, omega-3 supplement associated with ↑ mortality (HR = 1.73, 1.06–2.83; *p* = 0.029)	(−)
Vernieri et al., 2018 [81]	Review	Total *n* not reported Age not reported Male and female BMI not reported	All cancers All stages Therapy not specified	Not reported	Omega-3 supplement was tolerable with antitumor activity in 2 prospective trials for patients with advanced lung and breast cancer Preclinical study reported that the 16:4 (*n*-3) omega-3 in commercial fish oils impedes tumor-directed cytotoxicity of platinum compounds. Warns against indiscriminate fish oil supplementation	(+/−)

Abbreviations used: ^1^ Body Mass Index, ^2^ Randomized Controlled Trial, ^3^ Eicosapentanoic acid, ^4^ Docosahaxaenoic acid, ^5^ Brief Pain Inventory. The last column indicates the overall direction of the effects of Omega-3 supplementation on safety: (+) no risks to health; (−) some risks to health outcomes; (+/−) mixed risk profile. Relative risks (RR) and odds/hazard ratios (OR/HR) are shown as means with 95% confidence intervals.

**Table 5:** Safety of zinc supplements for patients with cancer.

Study	Type	Participants	Cancer	Dosage	Outcomes	Safety
De Sousa Melo et al., 2021 [82]	Narrative review	*n* not reported Age not indicated Male and female BMI ^1^ not reported	Head and neck cancer All stages Therapy: various	Varied	Zinc sulfate supplementation ↓ severity of mucositis, delayed its onset 25 mg/day ↓ incidence and duration of oral mucositis May induce nausea and vomiting, should not be taken on empty stomach	(+/−)

Abbreviations used: ^1^ Body Mass Index. The last column indicates the overall direction of the effects of Zinc supplementation on safety: (+) no risks to health; (−) some risks to health outcomes; (+/−) mixed risk profile. Relative risks (RR) and odds/hazard ratios (OR/HR) are shown as means with 95% confidence intervals.

## Supplementary Materials

The following are available online at https://www.mdpi.com/article/10.3390/cancers13236094/s1, Table S1. References supporting nutraceutical use against COVID-19.
