# Supplementary material for: A Narrative Review of the Safety of Anti-COVID-19 Nutraceuticals for Patients with Cancer
Source: Cancers (Basel). 2021 Dec 3;13(23):6094. doi: 10.3390/cancers13236094 (PMC8656592; doi:10.3390/cancers13236094)
Supplement: Supplementary file 1 [file cancers-13-06094-s001.zip › cancers-1485100-supplementary.pdf]

cancers MDPI

Supplementary Materials

Table S1. References supporting nutraceutical use against COVID-19.

Vitamin D
*N. Che Abdul Rahim, J. S. Manjit Singh, M. Pardi, A. A. Zainuddin, and R. Salleh, "Analysis of Available Nutrition Recommendations to Combat COVID-19: A Scoping Review," *Malays J Med Sci,* vol. 28, no. 3, pp. 18-45, Jun 2021, doi: 10.21315/mjms2021.28.3.3.
*S. Basak and J. Gokhale, "Immunity boosting nutraceuticals: Current trends and challenges," *J Food Biochem,* p. e13902, Sep 1 2021, doi: 10.1111/jfbc.13902.
*S. Corrao *et al.*, "Does Evidence Exist to Blunt Inflammatory Response by Nutraceutical Supplementation during COVID-19 Pandemic? An Overview of Systematic Reviews of Vitamin D, Vitamin C, Melatonin, and Zinc," *Nutrients,* vol. 13, no. 4, Apr 12 2021, doi: 10.3390/nu13041261.
*J. M. Evans *et al.*, "The Functional Medicine Approach to COVID-19: Virus-Specific Nutraceutical and Botanical Agents," *Integr Med (Encinitas),* vol. 19, no. Suppl 1, pp. 34-42, May 9 2020.
*G. Feketea, V. Vlacha, I. C. Bocsan, E. Vassilopoulou, L. A. Stanciu, and M. Zdrenghea, "Vitamin D in Corona Virus Disease 2019 (COVID-19) Related Multisystem Inflammatory Syndrome in Children (MIS-C)," *Front Immunol,* vol. 12, p. 648546, 2021, doi: 10.3389/fimmu.2021.648546.
*Z. Feng *et al.*, "Dietary supplements and herbal medicine for COVID-19: A systematic review of randomized control trials," *Clin Nutr ESPEN,* vol. 44, pp. 50-60, Aug 2021, doi: 10.1016/j.clnesp.2021.05.018.
*A. O. Ferreira, H. C. Polonini, and E. C. F. Dijkers, "Postulated Adjuvant Therapeutic Strategies for COVID-19," *J Pers Med,* vol. 10, no. 3, Aug 5 2020, doi: 10.3390/jpm10030080.
R. Lordan, H. M. Rando, C.-R. Consortium, and C. S. Greene, "Dietary Supplements and Nutraceuticals under Investigation for COVID-19 Prevention and Treatment," *mSystems,* vol. 6, no. 3, May 4 2021, doi: 10.1128/mSystems.00122-21.
F. Moscatelli *et al.*, "COVID-19: Role of Nutrition and Supplementation," *Nutrients,* vol. 13, no. 3, Mar 17 2021, doi: 10.3390/nu13030976.
G. F. Parisi *et al.*, "Nutraceuticals in the Prevention of Viral Infections, including COVID-19, among the Pediatric Population: A Review of the Literature," *Int J Mol Sci,* vol. 22, no. 5, Feb 28 2021, doi: 10.3390/ijms22052465.
E. Stachowska, M. Folwarski, D. Jamiol-Milc, D. Maciejewska, and K. Skonieczna-Zydecka, "Nutritional Support in Coronavirus 2019 Disease," *Medicina (Kaunas),* vol. 56, no. 6, Jun 12 2020, doi: 10.3390/medicina56060289.
C. W. Stratton, Y. W. Tang, and H. Lu, "Pathogenesis-directed therapy of 2019 novel coronavirus disease," *J Med Virol,* vol. 93, no. 3, pp. 1320-1342, Mar 2021, doi: 10.1002/jmv.26610.
L. Subedi, S. Tchen, B. P. Gaire, B. Hu, and K. Hu, "Adjunctive Nutraceutical Therapies for COVID-19," *Int J Mol Sci,* vol. 22, no. 4, Feb 16 2021, doi: 10.3390/ijms22041963.
R. Taha *et al.*, "The Relationship Between Vitamin D and Infections Including COVID-19: Any Hopes?," *Int J Gen Med,* vol. 14, pp. 3849-3870, 2021, doi: 10.2147/IJGM.S317421.
A. G. Vassiliou, E. Jahaj, S. E. Orfanos, I. Dimopoulou, and A. Kotanidou, "Vitamin D in infectious complications in critically ill patients with or without COVID-19," *Metabol Open,* vol. 11, p. 100106, Sep 2021, doi: 10.1016/j.metop.2021.100106.
M. X. Wang, S. X. W. Gwee, and J. Pang, "Micronutrients Deficiency, Supplementation and Novel Coronavirus Infections-A Systematic Review and Meta-Analysis," *Nutrients,* vol. 13, no. 5, May 10 2021, doi: 10.3390/nu13051589.
**Vitamin C**
*S. Basak and J. Gokhale, "Immunity boosting nutraceuticals: Current trends and challenges," *J Food Biochem,* p. e13902, Sep 1 2021, doi: 10.1111/jfbc.13902.
*N. Che Abdul Rahim, J. S. Manjit Singh, M. Pardi, A. A. Zainuddin, and R. Salleh, "Analysis of Available Nutrition Recommendations to Combat COVID-19: A Scoping Review," *Malays J Med Sci,* vol. 28, no. 3, pp. 18-45, Jun 2021, doi: 10.21315/mjms2021.28.3.3.
*J. M. Evans *et al.*, "The Functional Medicine Approach to COVID-19: Virus-Specific Nutraceutical and Botanical Agents," *Integr Med (Encinitas),* vol. 19, no. Suppl 1, pp. 34-42, May 9 2020.
*A. O. Ferreira, H. C. Polonini, and E. C. F. Dijkers, "Postulated Adjuvant Therapeutic Strategies for COVID-19," *J Pers Med,* vol. 10, no. 3, Aug 5 2020, doi: 10.3390/jpm10030080.
R. Lordan, H. M. Rando, C.-R. Consortium, and C. S. Greene, "Dietary Supplements and Nutraceuticals under Investigation for COVID-19 Prevention and Treatment," *mSystems,* vol. 6, no. 3, May 4 2021, doi: 10.1128/mSystems.00122-21.
G. F. Parisi *et al.*, "Nutraceuticals in the Prevention of Viral Infections, including COVID-19, among the Pediatric Population: A Review of the Literature," *Int J Mol Sci,* vol. 22, no. 5, Feb 28 2021, doi: 10.3390/ijms22052465.
L. Subedi, S. Tchen, B. P. Gaire, B. Hu, and K. Hu, "Adjunctive Nutraceutical Therapies for COVID-19," *Int J Mol Sci,* vol. 22, no. 4, Feb 16 2021, doi: 10.3390/ijms22041963.
**Selenium**
*S. Basak and J. Gokhale, "Immunity boosting nutraceuticals: Current trends and challenges," *J Food Biochem,* p. e13902, Sep 1 2021, doi: 10.1111/jfbc.13902.
*A. O. Ferreira, H. C. Polonini, and E. C. F. Dijkers, "Postulated Adjuvant Therapeutic Strategies for COVID-19," *J Pers Med,* vol. 10, no. 3, Aug 5 2020, doi: 10.3390/jpm10030080.
*Singh, 2020, Potential Inhibitors for SARS-CoV-2 and Functional Food Components as Nutritional Supplement for COVID-19: A Review
M. X. Wang, S. X. W. Gwee, and J. Pang, "Micronutrients Deficiency, Supplementation and Novel Coronavirus Infections-A Systematic Review and Meta-Analysis," *Nutrients,* vol. 13, no. 5, May 10 2021, doi: 10.3390/nu13051589.
**Omega 3 Fatty Acids**
*K. A. Adedokun *et al.*, "Addressing the global surge of COVID-19 cases: Insights from diagnostics, improved treatment strategies, vaccine development and application," *J Clin Transl Res,* vol. 7, no. 2, pp. 127-139, Apr 22 2021.

*S. Basak and J. Gokhale, "Immunity boosting nutraceuticals: Current trends and challenges," *J Food Biochem,* p. e13902, Sep 1 2021, doi: 10.1111/jfbc.13902.
* G. Derosa, P. Maffioli, A. D'Angelo, and F. Di Pierro, "Nutraceutical Approach to Preventing Coronavirus Disease 2019 and Related Complications," *Front Immunol,* vol. 12, p. 582556, 2021, doi: 10.3389/fimmu.2021.582556.
*A. O. Ferreira, H. C. Polonini, and E. C. F. Dijkers, "Postulated Adjuvant Therapeutic Strategies for COVID-19," *J Pers Med,* vol. 10, no. 3, Aug 5 2020, doi: 10.3390/jpm10030080.
R. Lordan, H. M. Rando, C.-R. Consortium, and C. S. Greene, "Dietary Supplements and Nutraceuticals under Investigation for COVID-19 Prevention and Treatment," *mSystems,* vol. 6, no. 3, May 4 2021, doi: 10.1128/mSystems.00122-21.
G. F. Parisi *et al.*, "Nutraceuticals in the Prevention of Viral Infections, including COVID-19, among the Pediatric Population: A Review of the Literature," *Int J Mol Sci,* vol. 22, no. 5, Feb 28 2021, doi: 10.3390/ijms22052465.
*Singh, 2020, Potential Inhibitors for SARS-CoV-2 and Functional Food Components as Nutritional Supplement for COVID-19: A Review
**Zinc**
*S. Basak and J. Gokhale, "Immunity boosting nutraceuticals: Current trends and challenges," *J Food Biochem,* p. e13902, Sep 1 2021, doi: 10.1111/jfbc.13902.
S. Corrao *et al.*, "Does Evidence Exist to Blunt Inflammatory Response by Nutraceutical Supplementation during COVID-19 Pandemic? An Overview of Systematic Reviews of Vitamin D, Vitamin C, Melatonin, and Zinc," *Nutrients,* vol. 13, no. 4, Apr 12 2021, doi: 10.3390/nu13041261.
*J. M. Evans *et al.*, "The Functional Medicine Approach to COVID-19: Virus-Specific Nutraceutical and Botanical Agents," *Integr Med (Encinitas),* vol. 19, no. Suppl 1, pp. 34-42, May 9 2020.
*Z. Feng *et al.*, "Dietary supplements and herbal medicine for COVID-19: A systematic review of randomized control trials," *Clin Nutr ESPEN,* vol. 44, pp. 50-60, Aug 2021, doi: 10.1016/j.clnesp.2021.05.018.
*A. O. Ferreira, H. C. Polonini, and E. C. F. Dijkers, "Postulated Adjuvant Therapeutic Strategies for COVID-19," *J Pers Med,* vol. 10, no. 3, Aug 5 2020, doi: 10.3390/jpm10030080.
*A. A. Oyagbemi *et al.*, "Potential health benefits of zinc supplementation for the management of COVID-19 pandemic," *J Food Biochem,* vol. 45, no. 2, p. e13604, Feb 2021, doi: 10.1111/jfbc.13604.
R. Lordan, H. M. Rando, C.-R. Consortium, and C. S. Greene, "Dietary Supplements and Nutraceuticals under Investigation for COVID-19 Prevention and Treatment," *mSystems,* vol. 6, no. 3, May 4 2021, doi: 10.1128/mSystems.00122-21.
F. Moscatelli *et al.*, "COVID-19: Role of Nutrition and Supplementation," *Nutrients,* vol. 13, no. 3, Mar 17 2021, doi: 10.3390/nu13030976.
G. F. Parisi *et al.*, "Nutraceuticals in the Prevention of Viral Infections, including COVID-19, among the Pediatric Population: A Review of the Literature," *Int J Mol Sci,* vol. 22, no. 5, Feb 28 2021, doi: 10.3390/ijms22052465.
P. Sharma, P. K. Reddy, and B. Kumar, "Trace Element Zinc, a Nature's Gift to Fight Unprecedented Global Pandemic COVID-19," *Biol Trace Elem Res,* vol. 199, no. 9, pp. 3213-3221, Sep 2021, doi: 10.1007/s12011-020-02462-8
*P. Singh, M. K. Tripathi, M. Yasir, R. Khare, M. K. Tripathi, and R. Shrivastava, "Potential Inhibitors for SARS-CoV-2 and Functional Food Components as Nutritional Supplement for COVID-19: A Review," *Plant Foods Hum Nutr,* vol. 75, no. 4, pp. 458-466, Dec 2020, doi: 10.1007/s11130-020-00861-9.
E. Stachowska, M. Folwarski, D. Jamiol-Milc, D. Maciejewska, and K. Skonieczna-Zydecka, "Nutritional Support in Coronavirus 2019 Disease," *Medicina (Kaunas),* vol. 56, no. 6, Jun 12 2020, doi: 10.3390/medicina56060289.
C. W. Stratton, Y. W. Tang, and H. Lu, "Pathogenesis-directed therapy of 2019 novel coronavirus disease," *J Med Virol,* vol. 93, no. 3, pp. 1320-1342, Mar 2021, doi: 10.1002/jmv.26610.
L. Subedi, S. Tchen, B. P. Gaire, B. Hu, and K. Hu, "Adjunctive Nutraceutical Therapies for COVID-19," *Int J Mol Sci,* vol. 22, no. 4, Feb 16 2021, doi: 10.3390/ijms22041963.
M. X. Wang, S. X. W. Gwee, and J. Pang, "Micronutrients Deficiency, Supplementation and Novel Coronavirus Infections-A Systematic Review and Meta-Analysis," *Nutrients,* vol. 13, no. 5, May 10 2021, doi: 10.3390/nu13051589.
**Quercetin**
*S. Basak and J. Gokhale, "Immunity boosting nutraceuticals: Current trends and challenges," *J Food Biochem,* p. e13902, Sep 1 2021, doi: 10.1111/jfbc.13902.
*J. M. Evans *et al.*, "The Functional Medicine Approach to COVID-19: Virus-Specific Nutraceutical and Botanical Agents," *Integr Med (Encinitas),* vol. 19, no. Suppl 1, pp. 34-42, May 9 2020.
G. F. Parisi *et al.*, "Nutraceuticals in the Prevention of Viral Infections, including COVID-19, among the Pediatric Population: A Review of the Literature," *Int J Mol Sci,* vol. 22, no. 5, Feb 28 2021, doi: 10.3390/ijms22052465.

*Support for use against COVID-19 mainly based on theoretical considerations.
